# A long terminal repeat retrotransposon of *Schizosaccharomyces japonicus* integrates upstream of RNA pol III transcribed genes

**DOI:** 10.1186/s13100-015-0048-2

**Published:** 2015-10-09

**Authors:** Yabin Guo, Parmit Kumar Singh, Henry L. Levin

**Affiliations:** Section on Eukaryotic Transposable Elements, Program in Cellular Regulation and Metabolism, Eunice Kennedy Shriver National Institute of Child Health and Human Development, National Institutes of Health, Building 18 T, room 106, Bethesda, MD 20892 USA; Present address: University of Texas Southwestern Medical Center, Dallas, Texas USA

**Keywords:** Tj1, Long terminal repeat retrotransposon, *Schizosaccharomyces japonicus*, *Schizosaccharomyces pombe*, Integration

## Abstract

**Background:**

Transposable elements (TEs) are common constituents of centromeres. However, it is not known what causes this relationship. *Schizosaccharomyces japonicus* contains 10 families of Long Terminal Repeat (LTR)-retrotransposons and these elements cluster in centromeres and telomeres. In the related yeast, *Schizosaccharomyces pombe* LTR-retrotransposons Tf1 and Tf2 are distributed in the promoter regions of RNA pol II transcribed genes. Sequence analysis of TEs indicates that Tj1 of *S. japonicus* is related to Tf1 and Tf2, and uses the same mechanism of self-primed reverse transcription. Thus, we wondered why these related retrotransposons localized in different regions of the genome.

**Results:**

To characterize the integration behavior of Tj1 we expressed it in *S. pombe*. We found Tj1 was active and capable of generating *de novo* integration in the chromosomes of *S. pombe.* The expression of Tj1 is similar to Type C retroviruses in that a stop codon at the end of Gag must be present for efficient integration. 17 inserts were sequenced, 13 occurred within 12 bp upstream of tRNA genes and 3 occurred at other RNA pol III transcribed genes. The link between Tj1 integration and RNA pol III transcription is reminiscent of Ty3, an LTR-retrotransposon of *Saccharomyces cerevisiae* that interacts with TFIIIB and integrates upstream of tRNA genes.

**Conclusion:**

The integration of Tj1 upstream of tRNA genes and the centromeric clustering of tRNA genes in *S. japonicus* demonstrate that the clustering of this TE in centromere sequences is due to a unique pattern of integration.

## Background

Centromeres contain a complex range of repeat DNA that typically contains clusters of transposable elements and repeat sequences derived from transposable elements [1–10]. The repeat nature of centromeres plays an important role in the assembly of heterochromatin and the formation of higher order structure that interacts with microtubules to mediate chromosome segregation [9, 11]. Although the clustering of transposable elements in and around centromeres occurs widely in eukaryotes from fungi to humans, it is not clear whether this relationship is due to the integration preferences of transposable elements or the accumulation of transposable elements in centromeres because there is little selection against them.

**Fig. 1:**
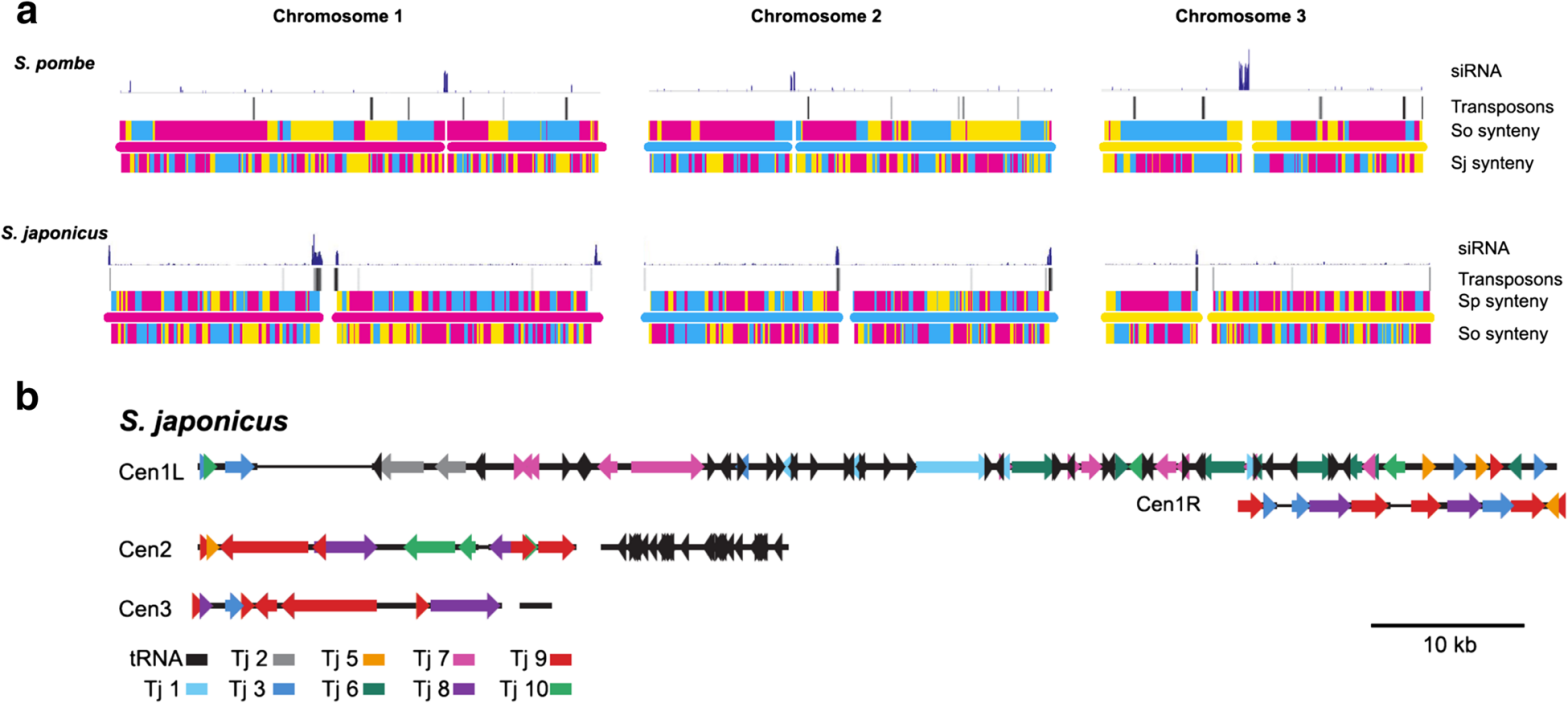
Tj1 elements and tRNA genes cluster in the centromeres of *S. japonicus.* **a** The 3 chromosomes of *S. pombe* and *S. japonicus* are shown with colors that indicate regions of synteny. So, Sj, and Sp are abbreviations for *Schizosaccharomyces octosporus, S. japonicus,* and *S. pombe,* respectively. The center bar in each chromosome is colored: red for chromosome 1, blue for chromosome 2, and yellow for chromosome 3. Transposable elements are mapped as black vertical lines and short RNA species previously detected are shown as purple vertical lines [6]. **b** Available contigs from the centromeres of *S. japonicus* are shown with colors that mark the positions of tRNA genes and different families of LTR-retrotransposons. Both **a** and **b** are reproduced from [6] reprinted with permission from AAAS

**Fig. 2:**
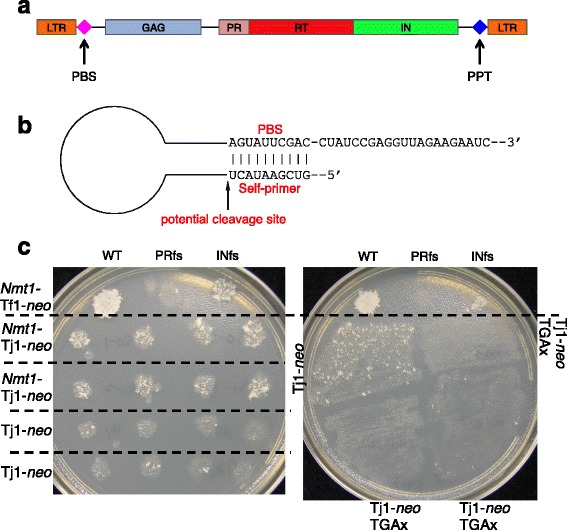
Tj1 is an active LTR-retrotransposon and likely relies of self-priming to initiate reverse transcription. **a** The structure of Tj1 is shown. Indicated are the positions of LTRs, and the coding sequences for Gag, PR, RT, and IN. The primer binding site (PBS) and the polypurine tract (PPT) are shown. **b** Sequence from the 5' LTR is shown indicating the upstream region that has complementarity to the PBS. We propose this serves as a self-primer of reverse transcription. Also shown is the potential cleavage site that is necessary to initiate priming of reverse transcription. **c** The FOA/G418 plates from the transposition assay. *Left Panel*: the top row shows transposition activity of *Nmt1*-Tf1-*neo* controls expressing wild type Tf1-*neo*, Tf1-*neo* with the protease frameshift (PRfs), and Tf1-*neo* with the integrase frameshift (INfs). Rows 2 and 3 each contain 4 independent transformants of *Nmt1*-Tj1-*neo*. Rows 4 and 5 each contain 4 transformants of Tj1-*neo* expressed from the Tj1 promoter. Growth of the patches is a measure of transposition frequency. *Right Panel*: The top row contains the *Nmt1*-Tf1-*neo* controls. In the second row the patch on the left contains Tj1-*neo*. The patch on the right side of the second row and the two patches on the 3^rd^ row are three independent transformants of Tj1-*neo* with a mutation that removes the stop codon at the end of Gag and as a result fuses all coding sequence into one ORF (Tf1-*neo* TGAx)

Whole genome sequencing of the fission yeast *Schizosaccharomyces japonicus* showed large numbers of transposable elements in the centromeres of all three chromosomes (Fig. 1a) [6]. Closer inspection of the centromere sequences showed clusters of tRNA genes were interspersed between the transposable elements (Fig. 1b) [6]. Analysis of the *S. japonicus* genome identified 13 full length long terminal repeat (LTR)-retrotransposons and 251 fragments of LTR-retrotransposons that together comprise 309 kb or 2.6 % of the 11.7 Mb genome of *S. japonicus* [6]. Phylogenetic analysis revealed that these transposable element sequences are derived from 10 distinct families of LTR-retrotransposons named Tj1 through Tj10 that are all members of the gypsy/Ty3 group of LTR-retrotransposons. One of the full-length LTR-retrotransposons belongs to the Tj1 family, is 5,003 bp in length, has two identical LTRs of 244 bp, has intact Gag and Pol open reading frames, and has an intact target site duplication of 5 bp on either side of the element (Fig. 2a). These properties suggest that this copy of Tj1 may be functional. Interestingly, the ORF for Gag possesses a stop codon upstream of Pol and the Pol ORF lacks an AUG codon. This configuration occurs in the type C retroviruses and functions as an unusual form of translational regulation that generates low levels of Pol proteins relative to Gag [12].

**Fig. 3:**
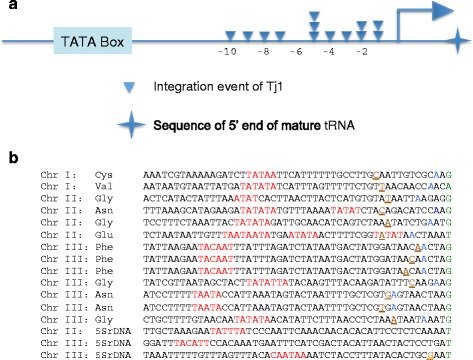
The positions of Tj1 integration clustered upstream of the TSSs of RNA pol III transcribed genes. **a** The region upstream of tRNA genes is diagramed showing the nucleotide positions of Tj1 integration. The TATA box is represented and the TSS is symbolized by the right-facing arrow. The star represents the sequence of the 5' end of the mature RNA species. While the linear representation is not drawn to scale the exact nucleotide positions of each integration site relative to the TSSs is shown. Some integrations mapped to the same tRNA gene but most occurred at different genes (Table 1). The two integrations downstream of the TSSs of 5S rRNA genes are not shown. **b** Sequences of the integration sites (Brown underlined) are shown upstream of the RNA pol III transcribed genes. Also shown are the putative TATA boxes (Red), the predicted TSSs (blue) and the 5' nucleotide encoding the 5' end of the mature RNA species (Green)

Another unusual property of Tj1 is the sequence of the primer-binding site (PBS) is complementary to the 5′ end of the Tj1 mRNA suggesting that Tj1 uses self-priming to initiate reverse transcription (Fig. 2b) [6]. Self-priming is a unique mechanism that instead of using a tRNA to prime reverse transcription relies on a 5′ segment of the mRNA from an LTR-retrotransposon to function as the primer. This mechanism was first demonstrated to occur for the LTR-retrotransposon Tf1 of *S. pombe* but has since been implicated in LTR-retrotransposons in hosts from fungi to vertebrates [13–19].

We decided to test Tj1 for transposition activity because it was one of the families of transposable elements that clustered in the centromeres of *S. japonicus* and determining insertion sites produced *de novo* would provide information about whether its integration occurred in centromere associated sequences. By expressing Tj1 marked with *neo* (Tj1-*neo*) in *Schizosaccharomyces pombe* we found the element was functional and readily produced integration events in chromosomal sites. The sequences of integration sites showed that Tj1 integrates upstream of RNA pol III transcribed genes. In the case of tRNA genes the inserts occurred between 1 and 10 bp upstream of predicted transcription start sites (TSSs). This integration bias together with the clusters of tRNA genes in the centromeres of *S. japonicus* indicates the clusters of Tj1 in centromere sequences resulted from the integration preference of Tj1.

## Results

One possible reason why there are clusters of LTR-retrotransposons in the centromeres of *S. japonicus* is that integration was targeted to these sequences. In order to test this possibility an active retrotransposon must be identified and *de novo* integration events mapped. To determine whether Tj1 possesses transposition activity we developed a genetic assay to detect Tj1 integration in *S. pombe.* We inserted *neo* upstream of the polypurine tract (PPT) in a full-length copy of Tj1 that was present in a high copy plasmid (Tj1-*neo*). We also constructed a version of this plasmid that removed the stop codon from the 3′ end of *gag*, resulting is one long ORF encoding all Tj1 proteins (Tj1-*neo* TGAx). A third plasmid was produced that expressed Tj1-*neo* from the *nmt1* promoter located on a plasmid (*Nmt1*-Tj1-*neo*). To detect transposition events *S. pombe* patches expressing the three versions of Tj1-*neo* were replica printed to medium containing 5-fluoroorotic acid (5FOA). This compound selects against the presence of the *URA3* gene in the expression plasmid. Cells lacking the Tj1-*neo* present in the plasmid must have integration events to be resistant to G418. In the final step of the assay the patches were replica printed to media containing 5FOA and G418 to test for the presence of integrated copies of Tj1-*neo* (Fig. 2c)*.* As a control for positive transposition we included cells expressing *Nmt1-*Tf1*-neo*, a highly active LTR-retrotransposon originally isolated from *S. pombe* [20–22]. The transposition assay of *Nmt1-*Tf1-*neo* produced high levels of resistance to G418 as previously described [15, 21, 22]. For a no transposition control we used *Nmt1-*Tf1-*neo* with a frame shift mutation at the N-terminus of IN (Tf1-*neo* INfs). This greatly reduced G418 resistance indicating as previously reported that the majority of the resistance produced by wild type *Nmt1*-Tf1-*neo* was due to integration events [15, 21, 22]. A frame shift in the N terminus of PR in *Nmt1-*Tf1-neo (Tf1-*neo* PRfs) produced little or no G418 resistance demonstrating that translation of PR and RT was required for the residual IN independent resistance of Tf1-*neo* INfs caused by homologous recombination between cDNA and pre-existing Tf sequences. Importantly, expression of *Nmt1*-Tj1-*neo* produced moderate levels of G418 resistance in eight independent transformants of *S. pombe* (Fig. 2c, left Panel)*.* Cells containing Tj1-*neo* showed lower levels of G418 resistance, indicating transposition frequencies were lower when Tj1 was expressed from its own promoter (Fig. 2c, left Panel). Cells expressing Tj1-*neo* TGAx showed virtually no resistance to G418, indicating that Tj1 proteins expressed as a single primary translation product were inactive. The G418 resistance of *Nmt1*-Tj1-*neo* and Tj1-*neo* indicated that Tj1 was capable of generating *de novo* integration events. The lack of activity of TGAx indicated that similar to Type C retroviruses, Tj1 function requires a stop codon at the end of Gag.

We determined the sequences of insertion sites by isolating genomic DNA from patches of G418^R^ cells produced by *Nmt1*-Tj1-*neo*. The DNA was fragmented by digestion with *Msp*I, ligated to a linker, and then insertion sites were amplified by PCR using primers specific for the Tj1 LTR and the linker (Methods). The sequences of PCR products identified 17 unique positions where Tj1 integrated (Table 1). We found 13 of the 17 integration sites occurred immediately upstream of tRNA genes. Three other integrations were near the 5′ end of 5S RNA genes showing that Tj1 had a strong preference for integration upstream of RNA pol III transcribed genes. One integration event occurred in the coding sequence of *exg2,* an RNA pol II transcribed gene. The integration sites upstream of tRNA genes were positioned between 5 and 11 bp upstream of the sequence encoding the mature 5′ ends of the RNAs (Table 1). However, the integrations at the 5S RNA genes were more broadly distributed and were located between 3 bp upstream to 41 bp downstream of the sequence encoding the mature 5′ end of the RNAs (Table 1).

**Table 1:** Position of Tj1 integration in *S. pombe*

Type	Gene Name	No. of inserts	Distance from sequence of mature RNA	Distance from transcription initiation site of RNA^a^	Orientation
tRNA	SPATRNAVAL.01	1	−11	−8	Forward
tRNA	SPATRNACYS.02	1	−11	−10	Forward
Gene	SPAC12B10.11^b^	1	1018	NA	Reverse
tRNA	SPBTRNAGLY.03	1	−10	−5	Forward
5S rRNA	SPRRNA.27	1	41	NA	Forward
tRNA	SPBTRNAASN.02	1	−11	−9	Forward
tRNA	SPBTRNAGLY.09	1	−10	−7	Forward
tRNA	SPBTRNAGLU.08	1	−11	−5	Forward
tRNA	SPCTRNAPHE.04	3	−5, −6, −7	−1, −2, −3	Forward
tRNA	SPCTRNAGLY.11	1	−6	−4	Forward
5S rRNA	SPRRNA.26	1	30	NA	Forward
tRNA	SPCTRNAASN.05	2	−11, 10	−1, −2	Forward
5S rRNA	SPRRNA.06	1	−3	NA	Forward
tRNA	SPCTRNAGLY.12	1	−9	−5	Forward

^a^Initiation sites were predicted based on location of TATA boxes

^b^RNA pol II transcribed gene

To map where integration occurred relative to RNA pol III transcription initiation we used positions of upstream TATA boxes to predict the locations of transcription initiation. Unlike the tRNA genes of *S. cerevisiae*, humans, and many other species, *S. pombe* tRNA genes have upstream TATA boxes that recruit TBP to sites where transcription initiates [23]. This analysis indicated that Tj1 integrated as close as 1, 2, and 3 bp upstream of the start of transcription (Fig. 3). The integration furthest from the start of transcription was 10 bp upstream of the TSS.

One surprising feature of all the integration events at the RNA pol III transcribed genes is that the Tj1 elements integrated in the same orientation relative to the target gene such that transcription of Tj1 was in the same direction as the RNA pol III gene (Table 1).

## Discussion

The identification of an intact LTR-retrotransposon from *S. japonicus* that is capable of inserting its sequence into chromosomal sites of the related fission yeast, *S. pombe,* offered the first opportunity to study the integration behavior of a transposable element from *S. japonicus.* Tj1-*neo* readily generated G418 resistance when expressed in *S. pombe*. The finding that the insertions had Tj1 LTR sequence that transitioned to *S. pombe* sequence at the first bp after the LTR provided strong evidence that Tj1-*neo* was active and capable of generating bona fide integration events. Our assays also revealed that the expression of Tj1 is similar to Type C retroviruses in that a stop codon at the end of Gag must be present for efficient integration.

Importantly, the positions of Tj1-*neo* integration showed a strong preference for sequences immediately upstream of RNA Pol III transcribed genes. None of the Pol III transcribed genes with integration were adjacent to centromere sequence indicating that Tj1 recognized Pol III transcription units not centromeres. This integration behavior is strikingly similar to the integration of the *Saccharomyces cerevisiae* LTR-retrotransposon Ty3 that integrates within a few bp of RNA Pol III transcription start sites [24–30]. The mechanism responsible for this integration has been determined by an elegant series of experiments demonstrating that portions of RNA Pol III transcription factor IIIB subunits, Brf1 and TBP interact with IN and Ty3 cDNA to position integration [31–33]. Whether Tj1 uses a similar mechanism will be the subject of future research. Thus far we were unable to identify amino acid similarities between the INs of Tj1 and Ty3 which could be important for interact with targeting factors.

The finding that Tj1 integrated with the same orientation as the transcription of the RNA pol III transcribed genes is unique. Although a truncation in a subunit of TFIIIC allows just one orientation of Ty3 integration, we know of no wild type LTR retrotransposon or retrovirus other than Tj1 that integrates with a fixed orientation relative to the transcription of target genes [34]. While this result indicates the interactions between Tj1 IN, its target sites, and its cDNA constrain the orientation of integration, it is formally possible that a selection existed that prevented the survival of integration in the opposite orientation.

The integration of Ty3 a few bp upstream of tRNA genes is a targeting mechanism that avoids disrupting coding sequence. In addition, the integration of Ty3 does not damage regulatory elements because the promoters of tRNA genes consist of A box and B box sequences that are within the transcribed sequences of the gene. However, recent studies of tRNA genes have shown in some species of *Schizosaccharomyces* such as *S. pombe* and *S. octosporus*, TATA box sequences are present between positions −20 and −35 bp upstream of the start of transcription [35]. Importantly, in *S. japonicus,* it appears that tRNA genes do not have upstream TATA boxes, indicating that Tj1 integration would not integrate between the promoter elements and the TSSs of tRNA genes [35]. Such an insertion would very likely disrupt the function of the promoters.

It is possible the clustering of LTR-retrotransposons in the centromeres of *S. japonicus* is the result of selective pressure either resulting from improved fitness of integration in these regions or due to reduced fitness associated with integration outside of centromeres. However, our finding that Tj1 integration occurs a few bp upstream of tRNA genes indicates that, at least in the case of Tj1, the transposable element sequences in the centromeres of *S. japonicus* are the result of the integration mechanism. This finding raises the possibility that integration of transposable elements in centromere regions is more general and that clustering of transposable elements in the centromeres of other eukaryotes could also be the result of integration bias. This possibility is supported by the observation that the LTR-retrotransposon Tal1 integrates with a strong bias for centromeric repeats of *Arabidopsis thaliana* [36]. Other evidence suggesting that integration into centromere sequences is more general includes the chromovirus lineage of LTR-retrotransposons [37–39]. The INs of these elements contain a chromodomain (CHD) that in proteins such as HP1 interacts with histone H3 methyl-K9 [40, 41]. When CHDs were identified in retrotransposon INs, it was suggested they play a role in target specificity [42, 43]. Subsequent experiments with three groups of chromoviruses showed that the CHDs of their INs direct subnuclear localization to foci coincident with heterochromatin [44]. The chromodomain of the fungal element, MAGGY, interacts with histone H3 dimethyl- and trimethyl-K9, and when the MAGGY chromodomain is fused to the IN of the Tf1 retrotransposon, new Tf1 insertions in *S. pombe* are directed to sites of H3 K9 methylation [44].

## Conclusions

Considerable evidence indicates that transposable elements contribute to the function of centromeres. Mutations that reactivate transposable elements in centromeres cause loss of heterochromatin which results is reduced binding of cohesin. This leads to reduced sister chromatid cohesion in *S. pombe* [45], loss of centromere condensation in *A. thaliana* [46], and chromosome segregation defects during meiosis in the mouse [47–49]. The question of how centromeres form requires an understanding of transposable elements and why they accumulate in centromeres. Although it is possible some transposable elements accumulate in centromere regions as the result of evolutionary selection, evidence is emerging that many transposable elements accumulate in centromeres because of integration patterns. Our finding that Tj1 integrates upstream of tRNA genes represents a unique strategy for concentrating transposable elements in the centromere. Compared with the LTR-retrotransposons that use CHDs to target centromeric heterochromatin [44], Tj1 integration is an evolutionary convergent method of localizing in centromeres and underscores the importance of the transposable elements in centromeres.

## Methods

### Media

Agar plates containing EMM were used to grow cells [50]. These plates were supplemented with 2 gm/l mixtures of an equal weight of all amino acids, 2.5 times more adenine with no uracil. Moreover, a final concentration of 10 μM thiamine was used to repress the transcription of Tj1 driven by the *nmt1* promoter. Further, EMM plates were supplemented by 1 g/l 5- fluoroorotic acid (5-FOA) and 50 mg/mL of uracil to eliminate the plasmid with Tj1-*neo*. Transposition was measured on plates that contained YES plus 1 g/l 5-FOA and 0.5 g/l G418 as previously described [15, 51].

### Plasmid construction, sample preparation, and sequencing

The sequence of Tj1 with annotations is available from Genbank with the accession number KT447435. The plasmids used in this study are listed in Table 2. Full-length Tj1 with *neo* upstream of the ppt was cloned by PCR from wild type *S. japonicus* (YHL9763) and inserted into the *Xho*I and *Bam*HI sites of pHL891 [16] to create pHL2848. pHL2851 was identical to pHL2848 except the stop codon at the 3′ end of *gag* was removed, resulting in a single ORF encoding all Tj1 proteins. To create pHL2861, the plasmid that expresses Tj1-*neo* from the *nmt1* promoter, we fused the self-priming sequence in the 5′ LTR that complements the PBS to the *nmt1* promoter. All Tj1 plasmids were transformed into *S. pombe* YHL912 (*h*^*−*^*, leu1-32, ura4-294*) for transposition assays.

**Table 2:** Plasmids

Plasmid	Description
pHL2848	Full-length copy of Tj1-*neo*
pHL2851	Full-length copy of Tj1-*neo*, stop codon between *gag* and *pol* removed
pHL2861	Tj1-*neo*, putative self-priming sequence in 5'-LTR fused to *nmt1* promoter

To generate the library of insertion sites genomic DNA was isolated from G418 resistant patches containing integration events. The genomic DNA was digested by *MspI* and a linker was ligated to the fragments. The linker ligated DNA fragments were digested with *Bam*HI to avoid amplifying the 5′ LTRs. The junction sequence downstream of the 3' LTR was amplified in PCR reactions that contained primers annealing to the LTR and the linker. PCR products were cloned in TOPO vector pCR2.1. These clones were used for Sanger sequencing.

### Mapping of Tj1 integration sites on the genome of *S. japonicus*

The sequence of the LTR and the linker were trimmed and the remaining sequences were used to find the coordinate of the insertion site by BLAST.

## Abbreviations

5FOA: 5-fluoroorotic acid
bp: Base pair
CHD: Chromodomain
mg: Milligram
ml: Milliliter
IN: Integrase
LTR: Long terminal repeat
PRfs: Protease frameshift
PBS: Primer binding site
INfs: Integrase frameshift
TSS: Transcription start site
